# CMTr mediated 2′-*O*-ribose methylation status of cap-adjacent nucleotides across animals

**DOI:** 10.1261/rna.079317.122

**Published:** 2022-10

**Authors:** Thomas C. Dix, Irmgard U. Haussmann, Sarah Brivio, Mohannakarthik P. Nallasivan, Yavor HadzHiev, Ferenc Müller, Berndt Müller, Jonathan Pettitt, Matthias Soller

**Affiliations:** 1 School of Biosciences, College of Life and Environmental Sciences, University of Birmingham, Edgbaston, Birmingham, B15 2TT, United Kingdom; 2 Birmingham Centre for Genome Biology, University of Birmingham, Edgbaston, Birmingham, B15 2TT, United Kingdom; 3 Department of Life Science, Faculty of Health, Education and Life Sciences, Birmingham City University, Birmingham, B15 3TN, United Kingdom; 4 Institute of Cancer and Genomic Sciences, College of Medical and Dental Sciences, University of Birmingham, Edgbaston, Birmingham, B15 2TT, United Kingdom; 5 School of Medicine, Medical Sciences and Nutrition, Institute of Medical Sciences, University of Aberdeen, Aberdeen, AB25 2ZD, United Kingdom

**Keywords:** mRNA methylation, 2′-*O*-ribose methylation

## Abstract

Cap methyltransferases (CMTrs) *O* methylate the 2′ position of the ribose (cOMe) of cap-adjacent nucleotides of animal, protist, and viral mRNAs. Animals generally have two CMTrs, whereas trypanosomes have three, and many viruses encode one in their genome. In the splice leader of mRNAs in trypanosomes, the first four nucleotides contain cOMe, but little is known about the status of cOMe in animals. Here, we show that cOMe is prominently present on the first two cap-adjacent nucleotides with species- and tissue-specific variations in *Caenorhabditis elegans*, honeybees, zebrafish, mouse, and human cell lines. In contrast, *Drosophila* contains cOMe primarily on the first cap-adjacent nucleotide. De novo RoseTTA modeling of CMTrs reveals close similarities of the overall structure and near identity for the catalytic tetrad, and for cap and cofactor binding for human, *Drosophila* and *C. elegans* CMTrs. Although viral CMTrs maintain the overall structure and catalytic tetrad, they have diverged in cap and cofactor binding. Consistent with the structural similarity, both CMTrs from *Drosophila* and humans methylate the first cap-adjacent nucleotide of an AGU consensus start. Because the second nucleotide is also methylated upon heat stress in *Drosophila*, these findings argue for regulated cOMe important for gene expression regulation.

## INTRODUCTION

Eukaryotic mRNAs contain a cap at their beginning that can be followed by variably methylated nucleotides. The main function of the cap is to protect mRNAs from degradation and to recruit translation initiation factors, but also to promote splicing and 3′ end processing (Topisirovic et al. 2011; Gonatopoulos-Pournatzis and Cowling 2014).

Caps are added cotranscriptionally in two steps shortly after transcription initiation of RNA polymerase II. First, a guanosine is added by RNGTT (RNA guanylyltransferase and 5′ phosphatase capping enzyme) in a characteristic 5′–5′ linkage to the first nucleotide of the mRNA and this guanosine is then methylated at the *N7* position by RNMT (RNA guanine-7 methyltransferase). Subsequently, the first two cap adjacent nucleotides can then be methylated at the ribose (2′-*O*-ribose methylation, cOMe) to various degrees between tissues and transcripts (Perry and Kelley 1974; Furuichi et al. 1975; Wei et al. 1975; Furuichi and Shatkin 2000; Galloway and Cowling 2019).

The cOMe mRNA modifications are introduced by dedicated cap methyltransferases (CMTrs) (Langberg and Moss 1981; Belanger et al. 2010; Werner et al. 2011). Most animals, including *Caenorhabditis elegans*, *Drosophila*, and mice model organisms as well as humans have two *CMTr* genes (*CMTr1* and *CMTr2*), whereas trypanosomes have three *CMTr* genes (Bangs et al. 1992). Moreover, many viruses including corona virus have their own *CMTr* gene (Ferron et al. 2012; Netzband and Pager 2020). Analysis of the first cap adjacent nucleotide has revealed variations in methylation between tissues and transcripts (Kruse et al. 2011; Mauer et al. 2017). Analysis of the second and following cap-adjacent nucleotides remains technically challenging (Anreiter et al. 2021). Initial studies on human CMTr activities had suggested that CMTr1 2′-*O*-ribose methylates the first and CMTr2 the second cap-adjacent nucleotide on a capped poly(A) substrate RNA or a transcript starting with three guanosines (Langberg and Moss 1981; Werner et al. 2011), but CMTr2 has also been shown to 2′-*O*-ribose methylate the second nucleotide of U1 or U2 snRNA starting with m7GpppAmpUpC (Werner et al. 2011). Mainly through the analysis of cOMe in trypanosomes, which is found on the first four nucleotides of the unique splice-leader trans-spliced onto each mRNA, it has been possible to analyze the contribution of individual CMTrs through knockouts (Arhin et al. 2006a,b; Zamudio et al. 2006, 2007). These studies suggested that CMTr1 would methylate the first nucleotide and CMTrs 2 and 3 methylate the following nucleotides of the unique splice leader added to mRNAs by trans-splicing, and these findings have been extrapolated to human CMTr1 and CMTr2 (Langberg and Moss 1981; Werner et al. 2011). However, recent findings in *Drosophila* using a novel recapping assay revealed that both CMTrs introduce cOMe on the first nucleotide redundantly. In addition, vaccinia CMTr VP39 can methylate up to the first three nucleotides of a trypanosome capped splice leader substrate in vitro (Haussmann et al. 2022).

In *Drosophila*, CMTr1 is the main enzyme and accounts for the majority of cOMe (Haussmann et al. 2022). CMTr1 in *Drosophila* and humans is nuclear, whereas CMTr2 localizes predominantly to the cytoplasm, but is also present in the nucleus and at cell membranes (Werner et al. 2011; Haussmann et al. 2022). Human CMTr1 interacts with the CTD of Pol II (Haline-Vaz et al. 2008). In *Drosophila* CMTr1 was shown to globally localize to sites of transcription, whereas CMTr2 only localizes to a subset of transcription sites suggesting divergent regulatory roles rather than constitutive functions in adding cOMe at a specific position in the beginning of the mRNA (Haussmann et al. 2022).

In addition to methylation introduced by CMTrs, when the first nucleotide of the mRNA is an adenosine and carries cOMe, it can also be methylated by PCIF1 in vertebrates and some other organisms. However, the mechanism for cap adenosine *N6*-methylation is different from internal methylation of adenosine (Keith et al. 1978; Kruse et al. 2011; Mauer et al. 2017; Akichika et al. 2019; Balacco and Soller 2019; Boulias et al. 2019; Sendinc et al. 2019; Sun et al. 2019; Pandey et al. 2020).

A high-resolution structure has been determined for the methyltransferase domain of human CMTr1 bound to a capped oligonucleotide and the methyl donor S-adenosylmethionine (SAM) (Smietanski et al. 2014). This structure has then served to model the methyltransferase domain of human CMTr2 bound to a capped oligonucleotide and SAM. In addition, structures of various viral CMTrs have been determined (Hodel et al. 1998; Malet et al. 2007; Zhao et al. 2015; Ferrero et al. 2019; Viswanathan et al. 2020). These structures reveal deep pockets for binding the cap and SAM. Because contacts between CMTr1 and cap-adjacent nucleotides are restricted to the RNA backbone, it is thought the 2′-*O*-ribose methylation occurs sequence independent.

Here, we applied a novel sensitive assay to analyse 2′-*O*-ribose methylation of cap-adjacent nucleotides beyond the first nucleotide to determine cOMe in various model organisms including *C. elegans*, *Drosophila*, honey bees, zebrafish, and mouse as well as human cell lines. This analysis reveals species- and tissue-specific differences in cOMe. In *Drosophila*, cOMe is mainly found on the first nucleotide. In contrast, in *C. elegans*, honey bees, zebrafish, mouse and human cell lines, cOMe is found on the first and second nucleotide. De novo structural modeling using RoseTTA structure prediction from human CMTr1 and viral CMTrs reveals overlapping configurations in the catalytic tetrad and binding of the cap structure, the RNA backbone and the methyl donor S-adenosylmethione (SAM) in animal CMTrs and trypanosome TbMTr1, but minor differences to viral CMTrs and trypanosome TbMTr2 and 3. Consistent with the structural similarity, both CMTrs from *Drosophila* and humans methylate the first cap-adjacent nucleotide of a capped substrate RNA starting with an AGU consensus observed for the majority of transcription start sites (Haussmann et al. 2022). Moreover, *Drosophila CMTr* double mutants are sensitive to heat stress, which can induce cOMe at the second position in *Drosophila* indicating that CMTrs can dynamically introduce cOMe at multiple positions in the first few nucleotides of an mRNA.

## RESULTS

### Cap-adjacent nucleotides are 2′-*O*-ribose methylated in a species- and tissue-specific manner

An alignment of CMTr1 and CMTr 2 orthologs of model animals and trypanososmes shows strong conservation of the overall domain structure and in the catalytic domain, but increasing differences outside these domains as expected from their phylogenetic divergence (Supplemental Figs. S1, S2; Werner et al. 2011). It has so far not been possible to directly and unambiguously determine the extent of 2′-*O*-methylation of cap-adjacent nucleotides in small amounts of mRNA. To close this technical gap, we have developed an assay based on recapping with ^32^P-αGTP followed by digestion with RNase I, which does not cleave in the presence of cOMe to allow for detection of consecutive cOMe on the first nucleotides of an mRNA by comparison to markers (note: in vitro transcribed RNA by bacterial polymerases requires a G or A as the first nucleotide, which will be a 5′ triphosphate and thus can be capped) (Haussmann et al. 2022).

**FIGURE 1.**
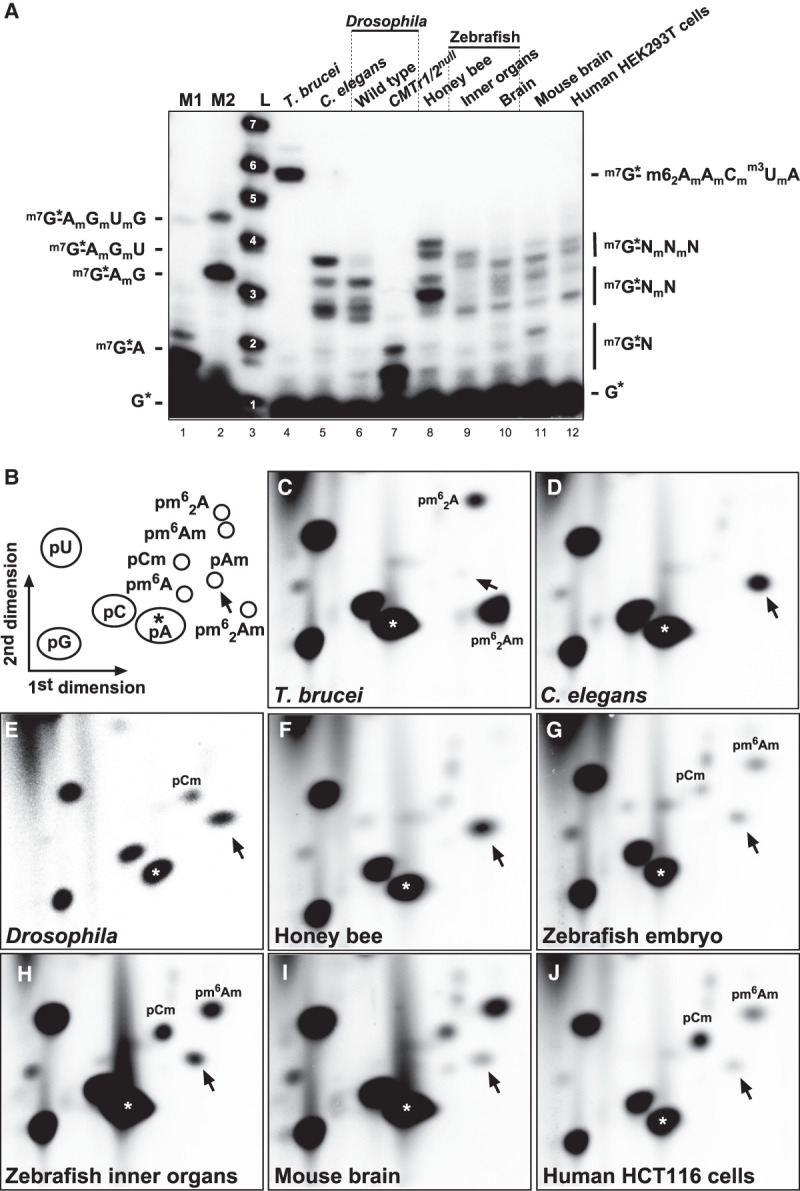
Analysis of mRNA cap 2′-*O*-ribose methylation in various species. (*A*) Recapping of mRNA with ^32^P-αGTP from Trypanosomes (*T. brucei*), adult *C. elegans*, adult *Drosophila* wild-type and CMTr1/2 double flies, worker honey bees, zebrafish inner organs and brain, mouse brain, and human HEK293T cells. 5′ cap structures were separated on a 22% denaturing polyacrylamide gels after digestion with RNase I (lanes *4*–*11*, *right*). Markers—M1: RNase I digested ^32^P-αGTP capped in vitro transcript starting with AGU. M2: RNase I digested ^32^P-αGTP capped in vitro transcript starting with AGU and 2′-*O*-ribose methylated with vaccinia CMTr. Sequences of markers are shown on the *left* and of cap structures from different species on the *right*, except for the sequence from Trypanososmes, which is shown on the *left*. L: Alkaline hydrolysis of a 5′ ^32^P-labeled RNA oligonucleotide with the nucleotide number indicated in white. (*B*) Schematic diagram of a 2D thin layer chromatography (TLC) depicting standard and 2′-*O*-ribose methylated nucleotides. For orientation, pA is indicated with an asterisk and pAm with an arrow. (*C*–*J*) TLCs showing modifications of the first cap-adjacent nucleotides of *T. brucei* (*C*), *C. elegans* (*D*), *Drosophila* (*E*), honey bees (*F*), zebrafish embryos and inner organs (*G*,*H*), mouse brain (*I*), and human HCT116 cells (*J*).

In the trypanosome *T. brucei*, we find a six nucleotide fragment consisting of the cap GTP, four 2′-*O*-ribose methylated nucleotides, and one nonmethylated nucleotide as previously reported by mass spectrometry analysis of the unique splice leader sequence, which is added to all mRNAs (Fig. 1A; Bangs et al. 1992; Arhin et al. 2006b; Zamudio et al. 2006). In *C. elegans*, we most prominently find cOMe on the first two cap adjacent nucleotides of mRNA at similar levels. *Drosophila* carries cOMe as previously reported only prominently on the first cap-adjacent nucleotide of mRNA, and cOMe is absent in *CMTr1/2* double mutants. In honey bees, zebrafish inner organs and brain, mouse brain and in human HEK293T cells, cOMe is also found prominently on the first two cap adjacent nucleotides at about equal amounts (Fig. 1A).

To specifically analyze methylation of the first nucleotide in poly(A) mRNA, we decapped and dephosphorylated poly(A) mRNA and labeled the first nucleotide by ^32^P-γATP. After digestion into individual nucleotides, they were separated on 2D thin layer chromatography (TLC) (Fig. 1B). In trypanosomes we detected *N6*-dimethylated adenosine that is also 2′-*O*-ribose methylated (pm^6^_2_Am, and to a lesser extent, *N6*-dimethylated adenosine (pm^6^_2_A, Fig. 1C) as previously reported (Bangs et al. 1992). In *C. elegans*, we detected cOMe prominently on adenosine (pAm) (Fig. 1D), but could not detect pGm present in about 70% of *trans*-spliced mRNAs starting with G in the splice leader (Allen et al. 2011; Pettitt et al. 2014), because pGm runs at the same position as pC (Haussmann et al. 2022). In *C. elegans*, pm^6^Am was absent in accordance with the absence of a *PCIF1* ortholog (Pandey et al. 2020), but pm^6^Am was also absent in *Drosophila* and honey bees (Fig. 1D–F). In Drosophila, the core catalytic sequence differs that could explain the absence, but this is not the case in honey bees (Supplemental Fig. S3).

In zebrafish embryos and inner organs, we detected pCm, pAm and m^6^Am (Fig. 1G,H). In the mouse brain, pm^6^Am is most prominent, followed by pCm and pAm (Fig. 1I). In human HCT116 cells, pCm is most prominent, followed by pm^6^Am and pAm (Fig. 1J).

### Eukaryotic and viral core CMTrs structures align in the catalytic center to bind the cap and SAM

**FIGURE 2.**
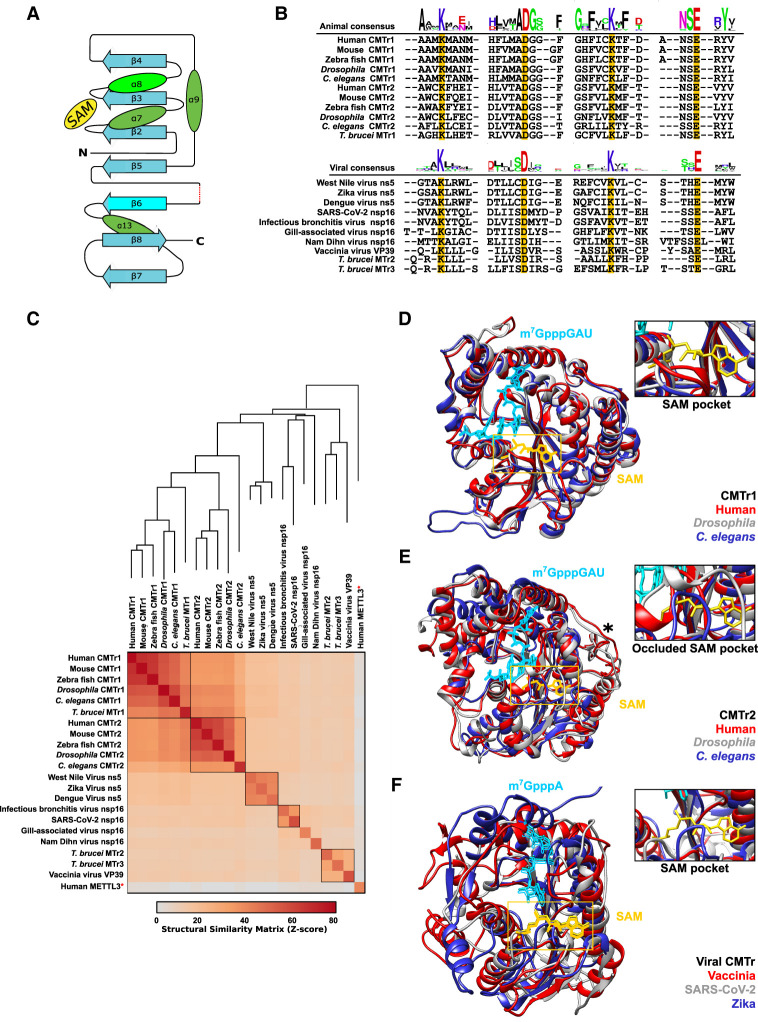
Structural comparisons of animal, protist, and viral CMTrs. (*A*) Schematic depiction of the Rossmann-like fold. (*B*) Structure-based multiple alignment of the sequence context surrounding the four key catalytic residues (yellow). The consensus for animal and viral CMTrs is shown on *top* and *bottom*, respectively. (*C*) Three-dimensional similarity of modeled CMTr structures determined by the DALI server depicting the structural similarity dendrogram on *top* and “all against all” multiple comparison at the *bottom* with orthologous proteins framed in black. Human Mettl3 (red star) was used as an outgroup indicating no similarity by near zero *z*-score (gray). (*D*,*E*) Three-dimensional structural alignment of CMTr1 (*D*) and CMTr2 (*E*) from humans (red), *Drosophila* (gray) and *C. elegans* (blue). The black star denotes the position of α 11 helix which is absent in *C. elegans*. The position of the cap analog (light blue) and the SAM (yellow) were inserted by superimposition from the published structure (PDB 4n48). (*F*) Three-dimensional structural alignment of CMTrs from vaccinia (red), SARS-CoV-2 (gray), and Zika virus (blue). The position of the cap analog (light blue) and the SAM (yellow) were inserted by superimposition from the published structure (PDB 1av6).

A key feature of methyltransferases is the characteristic Rossmann-like fold (Fig. 2A; Medvedev et al. 2021). To obtain insights into conserved features and the molecular mechanisms directing 2′-*O*-methylation of cap-adjacent nucleotides in diverse species we aligned the catalytic core from animal, protist, and viral CMTrs (Fig. 2B). This alignment reveals strict conservation of the four amino acids KDKE forming the catalytic tetrad (Lys, Asp, Lys, and Glu) and two major clades of an extended consensus surrounding the catalytic center for animal CMTrs including trypanosome TbMTr1 and viral CMTrs alongside similar trypanosome TbMTr2 and 3, respectively (Bujnicki and Rychlewski 2001; Feder et al. 2003; Zamudio et al. 2007).

Although the structure for human CMTr1 had been determined, extrapolating functional relationships from sequence alignments is limited because the sequence in more distantly related species such as *Drosophila* and *C. elegans* has diverged too much. Therefore, we used de novo structural prediction based on recently developed machine learning algorithms RoseTTA to compare the structures of CMTrs across model organisms, protists and viruses (Baek et al. 2021; Jumper et al. 2021). In particular, we built theoretical models of the methyltransferase (MTase) domain of both CMTr1 and CMTr2 from humans, mice, zebrafish, *Drosophila*, and *C. elegans*, as well as from the trypanosome *T. brucei* TbMTr1–3. In addition, we also modeled the MTase domain of viral CMTr nonstructural protein 16 (nsp16) from SARS-CoV-2, gill-associated virus (GAV), infectious bronchitis virus (IBV), and Nam Dihn virus (NDiV). Of note, the nonstructural protein 5 (ns5) from vaccinia (VP39), Zika, dengue, and West Nile viruses combines the entire capping process into one protein (Sutton et al. 2007).

To evaluate the accuracy of the predicted structures, we superimposed all models with corresponding structures available from the Protein Data Bank (PDB) for CMTr1 (4n48), SARS-CoV-2 nsp16 (6wk), and vaccinia VP39 (1av6). These structure-based pairwise alignments confirmed that they completely superimpose with root-mean-square deviation (RMSD) values, template modeling (TM), and global distance test (GDT) scores of 1.9/0.92/0.79 for CMTr1, 1.1/0.85/0.82 for VP39 and 1.1/96/0.92 for nsp16 (DALI server, Supplemental Fig. S4; Holm 2020).

To quantify the three-dimensional similarity of modeled CMTr structures, we performed an “all against all” comparative analysis via the DALI server of the MTase domain (Holm 2020). As an out-group control, we performed the same “all against all” analysis against the X-ray crystal structure of the human METTL3 (methyltransferase-like 3, 5il0) MTase domain, which also contains a Rossmann-like fold and catalyzes methylation of internal adenosine residues at the *N6* position (Wang et al. 2016; Balacco and Soller 2019).

As expected, the MTase domain of orthologous proteins framed in black clustered together with high structural similarity scores (*z* = 40–60, Fig. 2C) reminiscent of phylogenetic analysis from primary sequence alignment (Werner et al. 2011). Interestingly, trypanosome TbMTr1 is highly similar to eukaryotic CMTr1 (*z* = 39-44), whereas TbMTr2 and 3 are more closely related to vaccinia virus CMTr VP39 (*z* = 24, Fig. 2C). In contrast, the MTase domain of CMTrs compared to the METTL3 MTase domain had a very low structural similarity score (*z* < 5, Fig. 2C).

Superimposing the MTase domain of CMTr1 or CMTr2 from human, *Drosophila* and *C. elegans* revealed that they are highly similar in the core catalytic center and only diverged in peripheral parts by extended loops (Fig. 2D,E). In particular, the spatial position of the four amino acids forming the catalytic tetrad is near identical in the three animal species differing <1.5 Å (Supplemental Fig. S5A,B). Interestingly, CMTr2 contains an extended loop that occludes the SAM-binding pocket. Such a loop has also been found in SARS-CoV-2 nsp16, which opens upon association with nsp10 to allow binding of SAM (Vithani et al. 2021).

For the viral MTase domains from SARS-CoV-2 nsp 16, vaccinia VP39 and zika ns5, the core catalytic center also showed a high similarity, but peripheral parts are more diverged compared to animal CMTrs (Fig. 2F). Likewise, the four amino acids in the catalytic center adopt a similar position as in animal CMTrs differing <4 Å, but the first Lys of the catalytic tetrad shows more positional flexibility (Supplemental Fig. S5C).

Detailed analysis of the amino acids involved in cap and SAM binding in human, *Drosophila* and *C. elegans* CMTR1 reveals an essentially identical configuration (Supplemental Fig. S5D). Only one amino acid recognizing the amine group at position 2 of the cap guanosine changed from Asp to Ser in *C. elegans*. Other minor alterations include a change of Asn to Asp in recognizing the ribose of SAM in *Drosophila*. In CMTr2, although the amino acids involved in both cap and SAM binding have changed considerably between human, *Drosophila* and *C. elegans*, the contact positions are strictly conserved (Supplemental Fig. S5E). Likewise, viral CMTrs have substantially diverged to a nonoverlapping binding mode between vaccinia, Zika, and SARS-CoV-2. In addition, the cap is bound differently between animal CMTrs and viral CMTrs resulting in altered solvent exposure of the cap guanosine (Supplemental Fig. S5F).

### Human CMTr1 and CMTr2 structures align with trypanosome TbMTr1, and vaccinia VP39 with trypanosome TbMTr2 and 3, in the catalytic center to bind the cap and SAM

**FIGURE 3.**
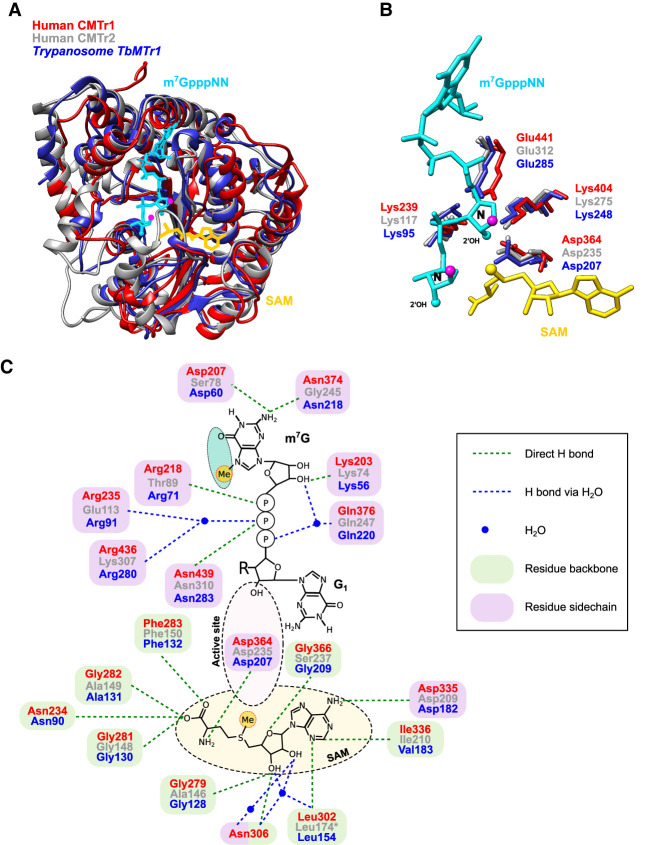
Comparison of human CMTr1 with human CMTr2 and trypanosome TbMTr1. (*A*) Superimposition of human CMTr1 (red), modeled human CMTr2 (gray), and trypanosome TbMTr1 (blue). Magenta circles indicate the base which has been removed for clarity. The position of the cap analog (light blue) and the SAM (yellow) were inserted by superimposition from the published structure (PDB 4n48). (*B*) Configuration of the four amino acids forming the catalytic center of human CMTr1 (red), modeled human CMTr2 (gray), and trypanosome TbMTr1 (blue). Magenta circles indicate the base which has been removed for clarity. (*C*) Substrate and cofactor recognition of human CMTr1 (red), modeled human CMTr2 (gray), and trypanosome TbMTr1 (blue). Amino acids contacting the cap binding are shown on *top* and amino acids contacting the cofactor SAM (yellow) are shown at *bottom*. The catalytic center is indicated by a dashed circle, and the turquoise circle indicates solvent facing. Contacts of amino acids via side chains are indicated by purple circles and via the backbone in green circles. Green and blue thin dashed lines indicate direct hydrogen bonds and hydrogen bonds via a water molecule, respectively. Magenta thick dashed lines indicate aromatic stacking. Methyl-groups are shown in yellow circles.

Superimposing the MTase domains of human CMTr1 and CMTr2 with the trypanososme TbMTr1 MTase domain shows that the overall structures are very similar, particularly in the core catalytic center and only diverged in peripheral parts (Fig. 3A). However, in CMTr2 an extended α helix protrudes into the catalytic site and SAM binding pocket suggesting structural rearrangement upon binding of either SAM or the cap. Again, the spatial position of the four amino acids forming the catalytic tetrad is near identical in human CMTr1 and 2, and trypanosome TbMTr1 differing <0.9 Å (Fig. 3B).

Detailed analysis of the amino acids involved in cap and SAM binding in human CMTr1 and CMTr2 compared to trypanosome TbMTr1 reveals essentially an identical configuration of how the cap and SAM are bound, although with variations in the contacting amino acids (Fig. 3C). Intriguingly, some amino acids altered in human CMTr2 compared to CMTr1 are the same in *Drosophila* CMTr2 compared to human CMTr1 such as the Asp contacting the amine group at position 2 of the cap guanosine and Arg contacting the γ phosphate in the 5′–5′ cap linkage. In the SAM binding pocket CMTr1 has two additional contacts at the carboxy group of methionine and at the ribose (Fig. 3C).

Vaccinia VP39 preferentially 2′-*O*-ribose methylates the first cap-adjacent nucleotide, but can extend the methylation to the second and even third nucleotide (Haussmann et al. 2022). Superimposing the MTase domains of vaccinia VP39 with trypanosome TbMTr2 and TbMTr3 MTase domains reveals principally an overall structure that is very similar, particularly in the core catalytic center, but TbMTr2 and TbMTr3 contain additional sequences in protruding loops above the substrate binding site (Supplemental Fig. S6A). Again, the spatial position of the four amino acids forming the catalytic tetrad is near identical in vaccinia VP39 compared to trypanosome TbMTr2 and TbMTr3 MTase domains differing <1.5 Å (Supplemental Fig. S6B). Likewise, the amino acids involved in cap and SAM binding in vaccinia VP39 and, trypanosome TbMTr2 and TbMTr3 MTase domains are identical for side chain interactions (Supplemental Fig. S6C).

Taken together, the high similarity of the different CMTr structures suggests that they will have very similar properties in adding cOMe to cap-adjacent nucleotides.

### Both human CMTrs 2′-*O*-ribose methylate the first and second cap-adjacent nucleotide

**FIGURE 4.**
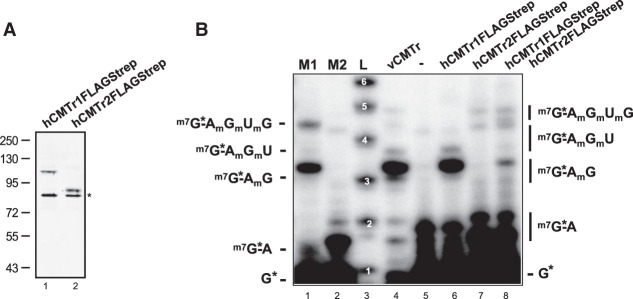
Analysis of mRNA cap 2′-*O*-ribose methylation in *C. elegans CMTr2* null mutants. (*A*) Representative western blot from two replicates of human CMTr1 and CMTr2 tagged with FLAGStrep expressed in human HEK293T cells detected with anti-FLAG antibodies. Molecular weight markers in kDa are shown on the *left*. The asterisk on the *right* denotes an unspecific band. (*B*) 2′-*O*-ribose methylation of a ^32^PαGTP capped in vitro transcript starting with AGU by human CMTR1 and CMTr2 expressed in human HEK293T cells, vaccinia CMTr (vCMTr) or extract from untransfected cells (−). 5′ cap structures were separated on a 22% denaturing polyacrylamide gels after digestion with RNase I (lanes *6*–*8*, *right*). Markers—M1: RNase I digested ^32^P-αGTP capped in vitro transcript starting with AGU. M2: RNase I digested ^32^P-αGTP capped in vitro transcript starting with AGU and 2′-*O*-ribose methylated with vaccinia CMTr. Sequences of cap structures are shown on the sides. L: Single nucleotide ladder with nucleotide number indicated in white.

Consistent with the highly similar structural models for CMTr1 and CMTr2, both can add cOMe to the first nucleotide in *Drosophila* (Haussmann et al. 2022). To test the activity of human CMTrs, we expressed them in human HEK293T cells (Fig. 4A) and incubated them with a capped AGU consensus starting substrate RNA.

In this assay, human CMTr1 primarily methylates the first, and to a low level also the second cap-adjacent nucleotide at the 2′ ribose position (Fig. 4B, lane 6), but no activity in the cell extract was detected (Fig. 4B, lane 5). In contrast, the main products detected after incubation of the substrate RNA with human CMTr2 in vitro contain 2′-*O*-ribose methylation on the second and third cap-adjacent nucleotide (Fig. 4B, lane 7), which is also evident for the vaccinia CMTr (Fig. 1A, lane 2 and Fig. 4B, lane 2). However, after the CMTr2 incubation 2′-*O*-ribose methylation of the first cap-adjacent nucleotide is not seen indicating that CMTr2 directly methylates the second cap-adjacent nucleotide once the first is methylated (Fig. 4B, lane 7). When human CMTr1 and CMTr2 were incubated together, cOMe on the second and third nucleotide increased (Fig. 4B, lane 8), but methylation with CMTr2 is slower than with CMTr1 because now also methylation on the first cap-adjacent nucleotide is detected (Fig. 4B, lane 8 compared to lane 7).

Taken together, both human CMTrs methylate the first cap-adjacent nucleotide as found in *Drosophila*, but CMTr2 more efficiently adds cOMe to the second and third cap-adjacent nucleotide preferably on a substrate containing cOMe on the first cap-adjacent nucleotide.

### *C. elegans* CMTr2 is redundant for 2′-*O*-ribose methylation of the second cap-adjacent nucleotide

To further evaluate the activity of CMTr1 in vivo, we used *C. elegans*, because both the first and second nucleotide carry cOMe. CMTr1 is well conserved in *C. elegans* (Supplemental Fig. S1), has the same methyltransferase domain structure, configuration of the catalytic tetrad, cap, and SAM binding as human and *Drosophila* CMTrs (Figs. 2, 3A).

**FIGURE 5.**
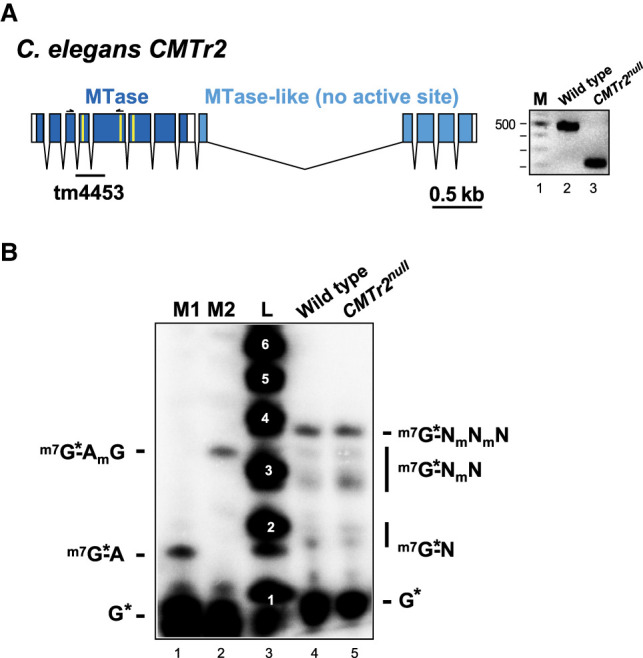
Analysis of mRNA cap 2′-*O*-ribose methylation in *C. elegans CMTr2* null mutants. (*A*) Genomic organization of the *C. elegans CMTr2* locus depicting the exon intron structure is shown on the *left*. The methyltransferase domain including key amino acid residues involved in catalysis (K117, D235, and K275 in yellow) is shown in dark blue and the catalytically inactive methyltransferase-like domain in light blue. The deletion in the *CMTr2* null allele (tm4453) leads to a frameshift and is validated by PCR products separated on an agarose gel on the *right*. M: 100 bp ladder. (*B*) Recapping of mRNA with ^32^P-αGTP from adult *C. elegans* wild-type and *CMTr2* null mutants. 5′ cap structures were separated on a 22% denaturing polyacrylamide gels after digestion with RNase I (lanes *4* and *5*, *right*) Markers—M1: RNase I digested ^32^P-αGTP capped in vitro transcript starting with AGU. M2: RNase I digested ^32^P-αGTP capped in vitro transcript starting with AGU and 2′-*O*-ribose methylated with vaccinia CMTr. Sequences of markers are shown on the *left* and of cap structures from *C. elegans* on the *right*. L: Alkaline hydrolysis of a 5′ ^32^P-labeled RNA oligonucleotide with the nucleotide number indicated in white.

A *CMTr2* null mutant in *C. elegans* harboring a small deletion inducing a frameshift in the catalytic domain is viable (Fig. 5A). In these mutants, the second cap-adjacent nucleotide still carries cOMe, indicating that CMTr1 and 2 can also act redundantly in adding cOMe to the second nucleotide in vivo (Fig. 5B).

### Elevated temperature reduces viability of *Drosophila CMTr1/2* double mutants and induces 2′-*O*-ribose methylation of the second cap-adjacent nucleotide

**FIGURE 6.**
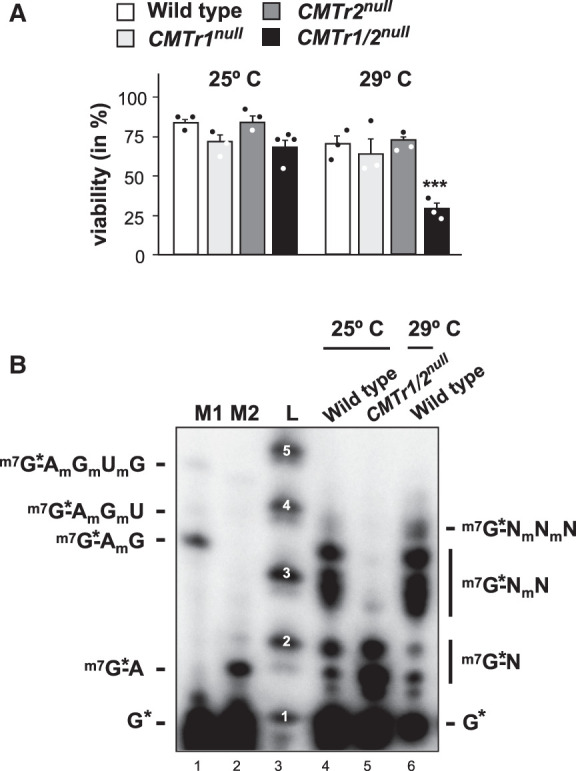
Impact of heat stress on *CMTr* double mutant viability and mRNA cap 2′-*O*-ribose methylation in *Drosophila*. (*A*) Viability of *CMTr1*^*null*^ and *CMTr2*^*null*^ single and double mutant flies at 25°C and 29°C shown as mean ± SE (*n* = 3, except for *CMTr1*/2^*null*^ at 25°C *n* = 4, *P* < 0.001). (*B*) Recapping of mRNA with ^32^P-αGTP from adult *Drosophila* wild-type and *CMTr* double mutants at 25°C and adult *Drosophila* wild-type flies kept at 29°C for 2 d. 5′ cap structures were separated on a 22% denaturing polyacrylamide gels after digestion with RNase I (lanes *4* and *5*, *right*) Markers—M1: RNase I digested ^32^P-αGTP capped in vitro transcript starting with AGU and 2′-*O*-ribose methylated with vaccinia CMTr. M2: RNase I digested ^32^P-αGTP capped in vitro transcript starting with AGU. Sequences of markers are shown on the *left* and of cap structures from *Drosophila* on the *right*. L: Alkaline hydrolysis of a 5′ ^32^P-labeled RNA oligonucleotide with the nucleotide number indicated in white.

Because methylation of mRNA has been linked to provide robustness to gene expression (Dezi et al. 2016; Haussmann et al. 2016; Roignant and Soller 2017), we wondered, whether loss of cOMe would reduce heat-tolerance of flies. When mutant flies of individual *CMTr1* and *CMTr2* genes were reared at 29°C, which is above the preferred temperature of 24°C (Sayeed and Benzer 1996), *CMTr1/2*^*null*^ double, but not single mutant flies were less tolerant to elevated temperature and showed significantly reduced survival (*P* < 0.001, Fig. 6A). These results again demonstrate redundancy of CMTrs in *Drosophila* (Haussmann et al. 2022).

We then tested whether acute increase in temperature for 2 d in adult flies would induce cOMe. Surprisingly, we now detected cOMe at the second cap-adjacent nucleotide in *Drosophila* in response to elevated temperature (Fig. 6B). However, when we reared *C. elegans* or *Drosophila* at low or high temperatures (15°C and 25°C for *C.elegans* and 18°C and 29°C for *Drosophila*) we did not detect differences in the extent of cOMe indicating no role of cOMe in temperature adaptation per se, but rather as a short-term response to stress (Supplemental Fig. S7).

## DISCUSSION

The most prominent and variable methylation of mRNA in animals and some of their parasites including trypanosomes and viruses such as SARS-CoV-2 is found at cap-adjacent nucleotides (Kruse et al. 2011; Mauer et al. 2017; Galloway and Cowling 2019). Intriguingly, both CMTrs in *Drosophila* add cOMe to the first nucleotide in vivo as shown by knockouts, as well as in vitro methylation assays (Haussmann et al. 2022). Here, we find that the ribose is variably 2′-*O*-methylated (cOMe) by CMTrs in a species and tissue-specific manner. In particular, the first nucleotide mostly contains cOMe, whereas cOMe on the second is variable among species and tissues with very little present in *Drosophila* and up to about half in other species. Likewise, if the first nucleotide is an adenosine, it is also variably methylated at the *N6* position (m^6^Am) by PCIF1 in vertebrates (Akichika et al. 2019). In *C. elegans*, the entire Mettl3 writer complex directing internal m^6^A and its YTH reader proteins are absent, and also PCIF1 is absent (Dezi et al. 2016; Balacco and Soller 2019), hence we did not detect m^6^Am on the first nucleotide in this species. In *Drosophila*, PCIF1 has a phenylalanine to histidine amino acid substitution in the NPPF catalytic center motif (Pandey et al. 2020), that could explain the absence of m^6^Am. In bees, we also did not detect m^6^Am, but the change of phenylalanine to tyrosine in the NPPF catalytic center motif unlikely explains the absence.

On the first nucleotide, cOMe is found most prominently at high levels of ∼80% or more in all species using a recapping assay, but is lower when using a decapping and relabeling assay (Haussmann et al. 2022). Several reasons could account for this difference. First, when preparing poly(A) RNA, non-cOMe containing RNA seems to copurify even after two rounds of poly(A) or ribo-minus selection. Also, incubation with the 5′–3′ exonuclease Xrn-1, which uses 5′ phosphorylated substrate, only marginally reduces this contaminant (Haussmann et al. 2022). Such RNA could be of various sources beyond degradation products and include un-capped sense and antisense RNAs and snoRNAs with complementarity to polyadenylated mRNA. Intriguingly, however, in both the TLC assays to analyze the first nucleotide and CAGEseq, adenosine was found to be the most frequent first nucleotide in *Drosophila* mRNA with little difference between the two methods (Haussmann et al. 2022). Second, using different decapping enzymes including tobacco acid pyrophosphatase, bacterial RppH, or mammalian decapping enzyme, we did not observe differences in the level of cOMe by labeling with T4 polynucleotide kinase indicating robustness of the assay, but also that these enzymes are not sensitive to mRNA secondary structure often found in the 5′ UTR. Whether the recently commercially available yeast yDcpS decapping enzyme is sensitive to secondary structure has not been extensively tested, but can be evaluated in the future when more CAGEseq data is becoming available from using yDcpS for library preparation (Wulf et al. 2019; Yan et al. 2022). Recently, also mass spectrometric analysis of caps in poly(A) mRNA has become possible, and these frequencies are similar to the recapping data (Akichika et al. 2019; Wang et al. 2019; Galloway et al. 2020).

In contrast to mass spectrometric analysis of caps in poly(A) mRNA, our recapping assay and separation on 22% denaturing acrylamide gels requires much less input material. Although this method is not able to identify the sequence of cap-adjacent nucleotides, it can accurately identify how many nucleotides adjacent to the cap are 2′-*O*-ribose methylated in mRNAs from wild-type or CMTr mutants, or in synthetic substrate RNAs by comparison to markers. The capped di-nucleotide of endogenous mRNA runs broader with about three identifiable bands as a result of sequence heterogeneity, while longer capped oligomers are merging into one band. We also noticed differences in ribose phosphate configurations, but the phosphatase activity of T4 PNK could not resolve this issue (Das and Shuman 2013).

Initial experiments characterizing CMTr activity using a capped poly(A) substrate and DEAE paper electrophoresis suggested preferential activity of CMTr1 for the first nucleotide, and CMTr2 for the second nucleotide (Langberg and Moss 1981). Similar results were also obtained with another unnatural capped RNA substrate starting with three guanosines (Werner et al. 2011). Although CMTr2 adds cOMe to U1 and U2 snRNA starting with AUA and AUC, whether it can also methylate the first nucleotide has not been tested (Werner et al. 2011). The cOMe status of mRNA has also been analyzed in trypanosome mutants for CMTrs using an RNase T2 digestion essay measuring cOMe by cleavage protection, because RNases require a nonmethylated 2′-hydroxyl group for catalysis (Motorin and Marchand 2018) and mRNAs contain a unique splice leader sequence (Bangs et al. 1992). Indeed, results from TbMTr2 and 3 mutants support a model whereby TbMTr1 would 2′-*O*-methylate the ribose of the first nucleotide and TbMTr2 and 3 the ribose of the three additional nucleotides (Arhin et al. 2006a,b; Zamudio et al. 2006, 2007). Possibly, this model specifically applies to trypanosomes, because of the unique sequence of the splice leader in combination with sequence specificity of TbMTrs (Mittra et al. 2008). Intriguingly, however, reinterpretation of the T2 digestion assay from TbMTr1 mutants suggest that an AmAmC fragment is present, indicating that TbMTr2 and 3 can add cOMe to the first and second nucleotide (Arhin et al. 2006a,b; Zamudio et al. 2006, 2007). Likewise, the vaccinia viral CMTr VP39, to which TbMTr2 and 3 are most closely related, adds cOMe to the first nucleotide, but can also methylate additional nucleotides (Haussmann et al. 2022). In addition, our previous genetic and biochemical studies of cOMe deposition in *Drosophila* showed that both CMTr1 and CMTr2 can methylate the first cap-adjacent nucleotide and that they act redundantly (Haussmann et al. 2022). Here, we further substantiate this view and show that also human CMTr2 can add cOMe to the first cap-adjacent nucleotide of a capped AGU consensus starting RNA substrate. Moreover, CMTr1 can also methylate the second nucleotide in a CMTr2 knockout in *C. elegans*, which are viable. In *Drosophila*, CMTr1 is the main enzyme as its absence reduces cOMe by about 80%. In addition, CMTr1 colocalizes with RNA Pol II to most if not all sites of transcription (Haussmann et al. 2022). Given the physical interaction of CMTr1 with RNA Pol II (Haline-Vaz et al. 2008), CMTr1 is likely also the main enzyme in other organisms, but because its loss is lethal in mice (Lee et al. 2020), this is more difficult to test.

From a structural point, the models of CMTr1 and 2 from humans, *Drosophila* and *C. elegans* are very similar in their overall methyltransferase structure, the catalytic tetrad and how the cap and SAM are bound, which is consistent with the capacity of both enzymes to add cOMe on both the first and second nucleotide. Although both enzymes only recognize the RNA backbone, but not the first nucleotide, there could be a preference for adding cOMe to the second nucleotide based on sequence context. In fact, such a scenario is suggested from the differences in the methylation pattern of vaccinia VP39 to a consensus AGU starting mRNA and the AACUAA starting trypanosome splice leader, which becomes more extensively 2′-*O*-ribose methylated (Haussmann et al. 2022). In this context, it is interesting that cOMe of the second nucleotide is most variable between different species and tissues. Hence, CMTr1 and 2 could differ in their activity in adding cOMe to the nucleotides after the first and impact differentially on gene expression this way. Moreover, developmental and cell-type specific roles are further suggested from the distinct expression profile of CMTrs in *Drosophila* (Haussmann et al. 2022). Likewise, CMTr1 is mostly nuclear whereas CMTr2 shows prominent cytoplasmic localization in both *Drosophila* and humans (Werner et al. 2011; Haussmann et al. 2022). In fact, a developmental role in *Drosophila* trachea development has specifically been attributed to CMTr2 suggesting sequence specificity, which has been found for trypanosome TbMTr1 (Englund et al. 1999; Mittra et al. 2008). Progress in the analysis of cOMe has been hampered by many technical challenges, but the combination of now available knockout models and the development of effective in vitro assay systems will allow to address substrate specificity in more detail in the future.

Taken together, our analysis of CMTrs across animals reveals redundant roles in adding cOMe to the first and second cap-adjacent nucleotide and potentially also to the third. Because transcription start sites are heterogeneous (Ohler et al. 2002) and such variability mediates a translational response to heat stress (Tamarkin-Ben-Harush et al. 2017), alterations in the methylation pattern of cap-adjacent nucleotides could essentially impact on gene expression regulating stability, localization, and translation.

## MATERIALS AND METHODS

### Animal husbandry and cell culture

*Caenorhabditis elegans* worms were kept on *E. coli* coated agar. The *CMTr2* mutant strain was generated by the National BioResource Project, which is part of the International *C. elegans* Gene Knockout Consortium and validated with PCR primers tm4453F (ATGATTTTGCCAGAAACCCGCG) and tm4453R (TGGTGCTTCCATCTGCAGTAAC).

Flies were kept on standard cornmeal-agar food (1% industrial-grade agar, 2.1% dried yeast, 8.6% dextrose, 9.7% cornmeal, and 0.25% Nipagin, all in w/v) in a 12 L: 12D cycle (Haussmann et al. 2013). CMTr mutants were as described (Haussmann et al. 2022). For the analysis of *Drosophila* survival at 29°C, flies were allowed to lay eggs on agar plates containing 1% grape juice and live yeast on top for 1 d at 25°C (Ustaoglu et al. 2019). After 48 h, larvae were washed off and collected on a fine mesh and the temperature shifted to 29°C. Groups of 30 larvae were transferred to food vials and flies were reared at designated temperatures. Honey bees were obtained and maintained as described (Decio et al. 2019; Decio et al. 2021).

Zebrafish were maintained in a designated facility (according to UK Home Office regulations) in a recirculating system (ZebTEC, Tecniplast) at 26°C in a 10-h dark, 14-h light photoperiod and fed three times daily. Animal work presented in this study was carried out under the project licences 40/3681 and P51AB7F76 assigned to the University of Birmingham. Inner organs were spleen, liver, and heart.

Mouse tissues were obtained from the Biomedical Service Unit of the University of Birmingham. Human HCT116 and HEK293T cells (ATCC) were cultured in McCoy's 5A and DMEM medium (Lonza) with 10% heat-inactivated FBS and 1% penicillin/streptomycin, respectively.

### Statistical analysis of behavioral data

Behavioral data was analyzed using GraphPad Prism. One-way ANOVA followed by a Tukey's post-hoc test was used for comparing multiple groups.

### Analysis of cap-adjacent 2′-*O*-ribose methylation

Total RNA was extracted with TRIzol (Invitrogen) and poly(A) mRNA was prepared by oligo dT selection according to the manufacturer (Promega). For the analysis of 5′ cap structures, 100 ng of poly(A) mRNA were decapped by yDcpS in 20 µL for 1 h at 37°C according to the manufacturer's instruction (NEB). Then the RNA was extracted by phenol/CHCl_3_ and ethanol precipitated in the presence of glycogen. The RNA was then labeled in a total volume of 20 µL containing 2 µL capping buffer (NEB), 1 µL SAM (2 mM), 0.25 µL ^32^P-αGTP (3000 Ci/mmol, 6.6 µM; Hartmann Analytics), 0.5 µL RNase Protector (Roche) and 0.5 µL capping enzyme (NEB) by incubation for 1 h at 37°C. The volume was then increased to 30 µL, and 54 µL AMPure XP magnetic beads (Beckman Colter) were added and the labeled mRNA purified according to the manufacturer's instructions. The RNA was then eluted in 10 µL DEPC treated water. A 2.5 µL aliquot was digested by adding 0.3 µL NEB buffer 2 and 0.3 µL RNase I for 2 h, and then 10 µL gel loading buffer was added (98% deionized formamide, 10 mM EDTA, 0.025% xylene cyanol FF and 0.025% bromphenol blue); products were analyzed on 22% denaturing polyacrylamide gels (National Diagnostics) and prerun for 2 h. Gels were soaked in 20% PEG400, 10% acetic acid and 40% methanol for 10 min and then dried on a Whatman 3MM paper. Dried gels were then exposed to a storage phosphor screen (Bio-Rad) and scanned by a Molecular Imager FX in combination with QuantityOne software (Bio-Rad). As a marker, a 31 nt in vitro transcript of the per gene made from a T7 2.5A promoter was capped and processed as described (Haussmann et al. 2022).

For the analysis of the first nucleotide in mRNA, poly(A) mRNA was purified from 30 µg of total RNA by oligo(dT) selection (Promega). Then, 50 ng of poly(A) mRNA was incubated with terminator nuclease (Epicenter) according to the manufacturer's instructions to remove rRNA followed by phenol/CHCl_3_ and ethanol precipitation in the presence of glycogen (Roche). The mRNA was then decapped using RppH (NEB) and dephosphorylated by Antarctic phosphatase (NEB) in NEB buffer 2 supplemented with 0.1 mM ZnCl_2_ in 20 µL. Then the RNA was extracted by phenol/CHCl_3_ and precipitated in the presence of glycogen. The 5′-ends of dephosphorylated mRNAs were then labeled using 10 units of T4 PNK (NEB) and 0.25 µL ^32^P-γATP (6000 Ci/mmol, 25 µM; Perkin-Elmer). The labeled RNA was precipitated, and resuspended in 10 µL of 50 mM sodium acetate buffer (pH 5.5) and digested with nuclease P1 (SIGMA) for 1 h at 37°C. Two microliters of each sample was loaded on cellulose F TLC plates (20 × 20 cm; Merck) and run in a solvent system of isobutyric acid:0.5 M NH_4_OH (5:3, v/v), as first dimension, and isopropanol:HCl:water (70:15:15, v/v/v), as the second dimension. TLCs were repeated from biological replicates. The identity of the nucleotide spots was determined as described (Keith 1995; Kruse et al. 2011). TLCs were exposed to a storage phosphor screen (Bio-Rad) and scanned by a Molecular Imager FX in combination with QuantityOne software (Bio-Rad).

Human CMTrs were cloned into a pSp plasmid containing a cytomegalovirus promoter using primers hCMTr1 F1 (CTGAAATCACTTTTTTTCAGGTTGGACCGGTGCCACCATGAAGAGGAGAACTGACCCAGAATGCACTGCC), hCMTr1 R1 (GTCATCGTCATCCTTGTAATCGCTCGAGGCCCTGTGCATCTGGATGAAGGAGAGGAC), hCMTr2 F1 (CTGAAATCACTTTTTTTCAGGTTGGACCGGTGCCACCATGAGTAAGTGCAGAAAGACACCAGTTCAG), and hCMTr2 R1 (GTCATCGTCATCCTTGTAATCGCTCGAGTTTTGTAACTGAAGGCTGTTGATAATTTCTTC), after reamplification with primer FLAG Strep R (GTTAGCAGACTTCCTCTGCCCTCGCTAGCCTTCTCGAACTGCGGGTGGGACCAGGCGCTCTTGTCATCGTCATCCTTGTAATC) to add a FLAG and a Strep tag to the C-term before the 2A peptide followed by a puromycin resistance gene. Proteins were then expressed in HEK293T cells after transfection with Transit2020 (Mirrus) reagent modified from the manufacturer's instructions. Briefly, 2 µL plasmid DNA (1 µg/µL) were mixed with 4 µL transfection regent and 600 µL serum-free DMEM media added and put on 1 Mio cells, grown in one well of a six-well plate, for 2.5 h before media was changed. After 1 d, puromycin (1:200, 10 mg/mL in water) was added and cells grown for 2 d. Cells were then lysed in 100 µL protein extraction buffer (20 mM Tris-HCL [pH 7.4], 137 mM NaCl, 1 mM EDTA, 25% glycerol [v/v], 1% NP-40 [v/v] 1 mM DTT, 1 mM PMSF, and protease inhibitor cocktail [Roche]).

To determine CMTr activity, 10 µL protein extract of CMTr1 was mixed with 10 µL reaction mix (20 mM Tris-HCL [pH 7.4], 0.2 µM SAM, 5 mM MgCl_2_, 2 mM DTT, 1 µL RNase Protector [Roche] and 1 µmole ^32^P-αGTP capped substrate RNA) and incubated for 30 min at room temperature. For CMTr2, 40 µL extract were mixed with 200 µL (20 mM Tris-HCL [pH 7.4], 137 mM NaCl) and 10 µL MAGStrep magnetic beads (type3 XT beads, IBA) and incubated for 2 h at 4°C. For the assay, 10 µL reaction mixture was added to the beads and incubated as described above. After phenol/CHCl_3_ extraction and ethanol precipitation in the presence of glycogen (Roche), the RNA was taken up in 10 µL of water. Then, 0.3 µL NEB buffer 2 and 0.3 µL RNase I (NEB) was added to 2.5 µL RNA and digested for 2 h. Then 10 µL gel loading solution was added and analyzed on 22% gels as described above.

As substrate RNA, a 600 nt RNA starting with 31 nt from the *Drosophila* per gene containing a AGU consensus start was used (Haussmann et al. 2022), which was transcribed with T7 polymerase from a EcoRI and Spe I linearized plasmid in a 100 µL reaction using the Ambion MEGAscript kit (Ustaoglu et al. 2021). Six micrograms of RNA (3 µL, 11 µM) was then labeled with 3 µL ^32^P-αGTP in 20 µL as described above and 1 µL was used per CMTr labeling reaction.

### Structural modeling

Alignments of CMTrs were done by ClustalW and conservative substitution determined by BLOSUM-62. Full-length amino acid sequences were used for theoretical modeling via the Robetta protein structure prediction service using the RoseTTA deep learning server (Baek et al. 2021). The theoretical models of CMTrs were “trimmed” to the minimal methyltransferase catalytically active unit and written to a new PDB file. This region is indicated on the CMTr1 and 2 alignments (Supplemental Figs. S1, S2).

### Quality assessment of theoretical protein structures

The quality of the theoretical models was determined by error-coverage plots determined by the RoseTTA algorithm on the Robetta server (Baek et al. 2021). In addition, theoretical models were superimposed with their corresponding experimentally determined protein structures hMTr1 (4n48), SARS-CoV-2 nsp16 (6 wk), and Vaccinia virus VP39 (1av6) (Hodel et al. 1998; Smietanski et al. 2014; Viswanathan et al. 2020). For quantitative comparison, the theoretical model and corresponding X-ray crystal structure were subjected to DALI server pairwise analysis to determine average RMSDs for all common residues and a structural similarity *z*-score (Holm 2020). Moreover, the template modeling (TM) and global distance test (GDT) score was determined via the TM-score server (Xu and Zhang 2010). The DALI server was used for “all against all” protein structure comparison (Holm 2020). Protein structure visualization, superimposition, and determination of Cartesian coordinates were performed using the UCSF Chimera package downloaded from the Resource for Biocomputing, Visualization, and Informatics at the University of California (Pettersen et al. 2004).

## SUPPLEMENTAL MATERIAL

Supplemental material is available for this article.

## Supplementary Material

Supplemental Material

## Figures and Tables

**Thomas Dix:**
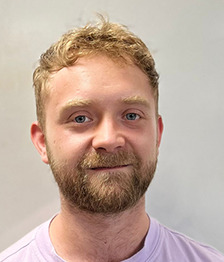


**Irmgard Haussmann:**
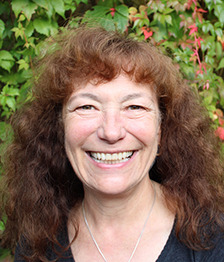


## MEET THE FIRST AUTHORS

***Meet the First Author(s)* is a new editorial feature within *RNA*, in which the first author(s) of research-based papers in each issue have the opportunity to introduce themselves and their work to readers of *RNA* and the RNA research community. Thomas Dix and Irmgard Haussmann are the co-first authors of this paper, “CMTr mediated 2′-*O*-ribose methylation status of cap adjacent-nucleotides across animals.” Tom is a BBSRC-funded post-doctoral research fellow in Matthias Soller's laboratory at the University of Birmingham, UK. His current research revolves around the mechanisms of alternative splicing in *Drosophila* and how mRNA methylation affects differential mRNA processing. Irmi is an associate professor in health science at Birmingham City University. She started working with RNA during her PhD, working on U7 snRNA processing in the laboratory of Professor Daniel Schuemperli at the University of Berne, Switzerland, and has a continued interest in mRNA and post-transcriptional gene regulation.**

**What are the major results described in your paper and how do they impact this branch of the field?**

We used de novo RoseTTA modeling of CMTr structures and found that animal CMTrs are all very similar in their catalytic domain, which helps to explain redundancies in enzymatic assays and in animal models. We have developed a new assay to look at the extent of methylation of the first few nucleotides in very little mRNA of various animals and human cell lines. Using this assay, we surprisingly found that *Drosophila* mainly methylates the first cap-adjacent nucleotide while other animals mostly methylate the first two.

**What led you to study RNA or this aspect of RNA science?**

**TD:** Since my bachelors, I have been fascinated by how humans can make more than 200,000 distinct proteins from just about 20,000 genes by differential mRNA processing. I'm particularly intrigued by how genes can be alternatively spliced and how mRNA methylation impacts on gene expression.

**IH:** Methylation of cap-adjacent nucleotides has been known since the seventies, but the enzymes responsible for this modification have only been discovered 10 years ago. Still, we know very little about the biological function of these major RNA modifications.

**During the course of these experiments, were there any surprising results or particular difficulties that altered your thinking and subsequent focus?**

**TD:** Due to a major story coming out of the laboratory about CMTrs and my inherent interest in structural biology, I became interested in comparing the structures of human and viral CMTrs during the first UK lockdown, as I was no longer allowed to leave my house. This ultimately laid the foundation of the work presented here.

**IH:** Since starting this project 10 years ago with generating *Drosophila* knockouts, this project has taken many twists and turns, and looking at these modifications remains technically very challenging.

**What are some of the landmark moments that provoked your interest in science or your development as a scientist?**

**TD:** During my bachelors at Coventry University, I became interested in the mechanisms behind mRNA processing due to the enthusiastic discussions with Irmi, at the time my undergraduate dissertation supervisor.

**IH:** When starting my research career, it became obvious that gene expression can be regulated in so many ways at the level of RNA and I'm sure many fundamental discoveries remain to be made.

**If you were able to give one piece of advice to your younger self, what would that be?**

**IH:** No matter how difficult your journey is, keep going. It is the journey and people you interact with that make the memories.

**Are there specific individuals or groups who have influenced your philosophy or approach to science?**

**IH:** At my first RNA meeting as a young PhD student in Marburg, Germany, I met all the famous PIs in the RNA field. I was inspired by their ideas and aspirations, and surprised at how wild the parties were.

**What are your subsequent near- or long-term career plans?**

**IH:** With my increasing teaching and administration tasks I am keen to find time for research during semester breaks.

**What were the strongest aspects of your collaboration as co-first authors?**

We teamed up with complementary skills and expertise to approach the topic. It was fantastic to see how the different approaches of molecular and structural biology came together.
