# Supplementary material for: CMTr mediated 2′-O-ribose methylation status of cap-adjacent nucleotides across animals
Source: RNA. 2022 Oct;28(10):1377–90. doi: 10.1261/rna.079317.122 (PMC9479742; doi:10.1261/rna.079317.122)
Supplement: Supplemental Material [file supp_28_10_1377__DC1.html]

CMTr mediated 2`-O-ribose methylation status of cap adjacent-nucleotides across animals — CMTr mediated 2′-O-ribose methylation status of cap-adjacent nucleotides across animals — Supplemental Material 

# CMTr mediated 2′-*O*-ribose methylation status of cap-adjacent nucleotides across animals

## Supplemental Material

- Supplemental\_Material.pdf
