# Supplementary material for: CMTr mediated 2′-O-ribose methylation status of cap-adjacent nucleotides across animals
Source: RNA. 2022 Oct;28(10):1377–90. doi: 10.1261/rna.079317.122 (PMC9479742; doi:10.1261/rna.079317.122)

CMTr mediated 2`-*O*-ribose methylation status of cap adjacent-nucleotides across animals

THOMAS C. DIX[1,2*], IRMGARD U. HAUSSMANN[3*], SARAH BRIVIO[4], MOHANNAKARTHIK P. NALLASIVAN[1,2], YAVOR HADZHIEV[2,5], FERENC MÜLLER[2,5], BERNDT MÜLLER[4], JONATHAN PETTITT[4] AND MATTHIAS SOLLER[1,2,6]

Figure legends for Supplemental Figures

Supplemental Figure S1. Alignment of CMTr1 from human, mouse, zebrafish, *Drosophila*, *C. elegans* and trypanosome TbMTr1. The domain structure is indicated on top of the sequence. Identical and conserved amino acids are indicated in red and orange, respectively. Blue arrows indicate the amino acids of the catalytic tetrad and red arrows the boundary for the methyltransferase domain used for modelling.

Supplemental Figure S2. Alignment of CMTr2 from human, mouse, zebrafish, *Drosophila* and *C. elegans*. The domain structure is indicated on top of the sequence. Identical and conserved amino acids are indicated in red and orange, respectively. Blue arrows indicate the amino acids of the catalytic tetrad and red arrows the boundary for the methyltransferase domain used for modelling.

Supplemental Figure S3. Alignment of PCIF1 from human, mouse, zebrafish, *Drosophila*, *C. elegans* and *T. brucei*. The domain structure is indicated on top of the sequence. Identical and conserved amino acids are indicated in red and orange, respectively. Pink arrows indicate amino acids in the catalytic centre important for catalytic activity and green arrows amino acids involved in cap binding.

Supplemental Figure S4. Validation of modelled structures. Superimposed structures for human CMTr1 (A), vaccinia virus VP39 (B) and SARS-CoV-2 nsp16 (C) are shown on the left with the X-ray structure in orange, the modelled structure in magenta, the cap analogue in light blue and SAM in yellow. On the right side the error estimate (in Angstrom) is plotted for each residue with the methyltransferase domain indicated in yellow. SARS-CoV-2 nsp16 was co-crystallized with its bound co-factor nsp10.

Supplemental Figure S5. Structural comparison of animal CMTr1, CMTr2 and viral CMTr tetrad configuration, cap and SAM binding.

(A-C) Configuration of the four amino acids forming the catalytic centre of CMTr1 and CMTr2 for human (red), *Drosophila* (grey) and *C. elegans* (blue) and viral CMTr for Vaccinia VP39 (red), Zika ns5 (grey) and SARS-CoV-2 nsp16 (blue), respectively. SAH: S-adenosylhomocystein (orange). The position of the cap analogue (light blue) and the SAM (yellow) or SAH (orange) were inserted by superimposition from the published structure (PDB 4n48 in A and B, and PDB 6wks for nsp16 and PDB 1av6 for VP39 and ns5 with the cap analogue in white for VP39 in C).

(D-F) Substrate and co-factor recognition of CMTr1 and CMTr2 for human (red), *Drosophila* (grey) and *C. elegans* (blue) and viral CMTr for Vaccinia VP39 (red), Zika ns5 (grey) and SARS-CoV-2 nsp16 (blue), respectively. Amino acids contacting the cap binding are shown on top and amino acids contacting the co-factor SAM (yellow) are shown at the bottom. The catalytic centre is indicated by a dashed circle and the turquoise circle indicates solvent facing. Contacts of amino acids via side chains are indicated by or purple circles and via the backbone in green circles. Green and blue thin dashed lines indicate direct hydrogen bonds and hydrogen bonds via a water molecule, respectively. Magenta thick dashed lines indicate aromatic stacking. Methyl-groups are shown in yellow circles.

Supplemental Figure S6. Comparison of vaccinia VP39 structure with trypanosome TbMTr2 and TbMTR3.

(A) Superimposition of vaccinia VP39 (red), modelled trypanosome TbMTr2 (grey) and TbMTr3 (blue). The position of the cap analogue (light blue) and the SAH (yellow) were inserted by superimposition from the published structure (PDB 1av6).

(B) Configuration of the four amino acids forming the catalytic centre of vaccinia VP39 (red), modelled trypanosome TbMTr2 (grey) and TbMTr3 (blue). SAH: S-adenosylhomocystein.

(C) Substrate and co-factor recognition of vaccinia VP39 (red), modelled trypanosome TbMTr2 (grey) and TbMTr3 (blue). Amino acids contacting the cap binding are shown on top and amino acids contacting the co-factor SAH (yellow) are shown at the bottom. The catalytic centre is indicated by a dashed circle and the turquoise circle indicates solvent facing. Contacts of amino acids via side chains are indicated by or purple circles and via the backbone in green circles. Green and blue thin dashed lines indicate direct hydrogen bonds and hydrogen bonds via a water molecule, respectively. Magenta thick dashed lines indicate aromatic stacking. Methyl-groups are shown in yellow circles.

Supplemental Figure S7. mRNA cap 2`-*O*-ribose methylation is not used for temperature adaptation in *C. elegans* and *Drosophila*.

Recapping of mRNA with ^{32}P-alphaGTP from *C. elegans* and *Drosophila* reared at low and high temperature (15ºC and 25ºC for *C.elegans* and 18ºC and 29ºC for *Drosophila*). Markers – M1: RNAse I digested ^{32}P-alphaGTP capped *in vitro* transcript starting with AGU. M2: RNAse I digested ^{32}P-alphaGTP capped *in vitro* transcript starting with AGU and 2'-*O*-ribose methylated with vaccinia CMTr. Sequences of markers are shown on the left and of cap structures are shown on the right. L: Alkaline hydrolysis of a 5' ^{32}P-labeled RNA oligonucleotide with the nucleotide number indicated in white.

Supplemental Figure S1

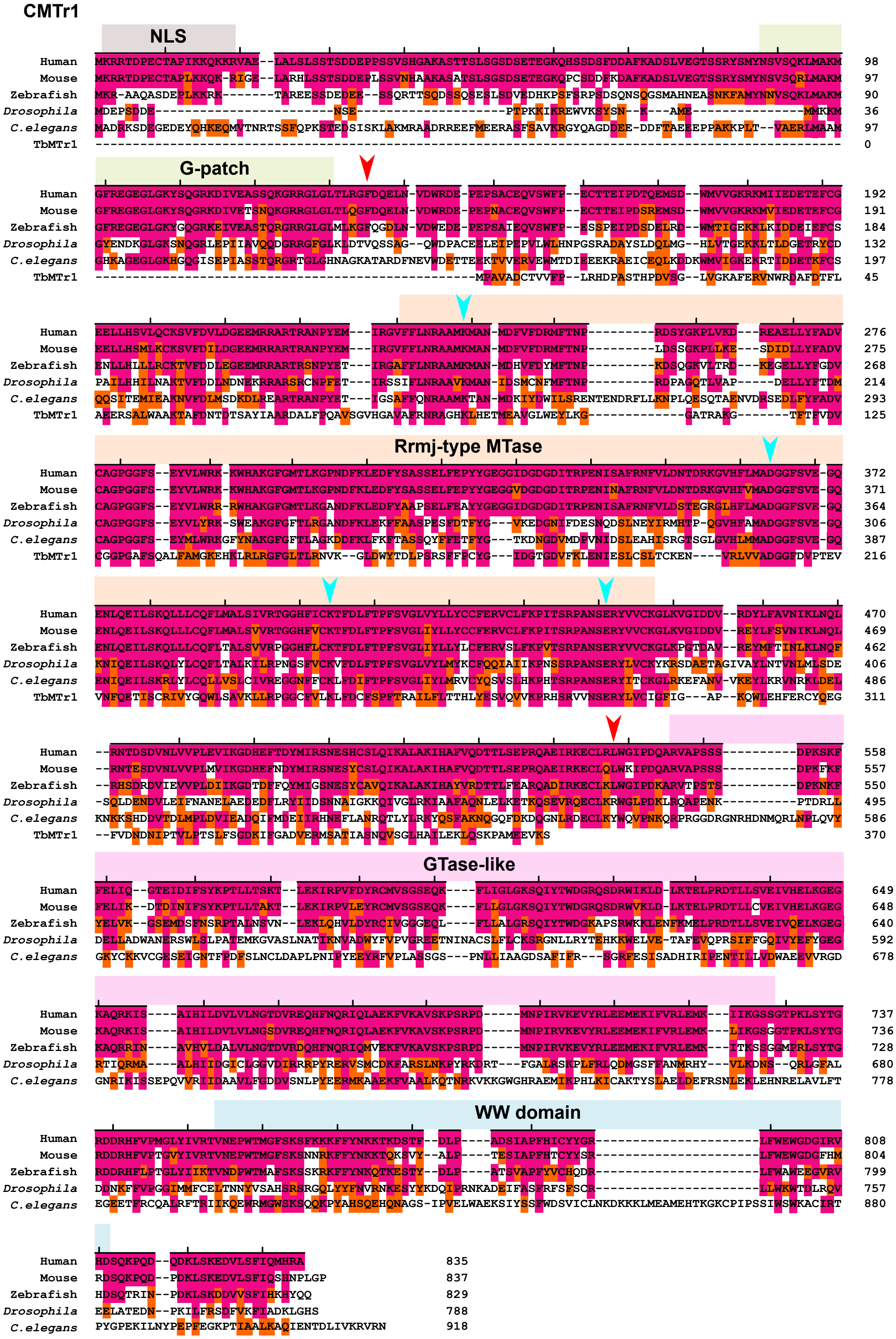

Supplemental Figure S2

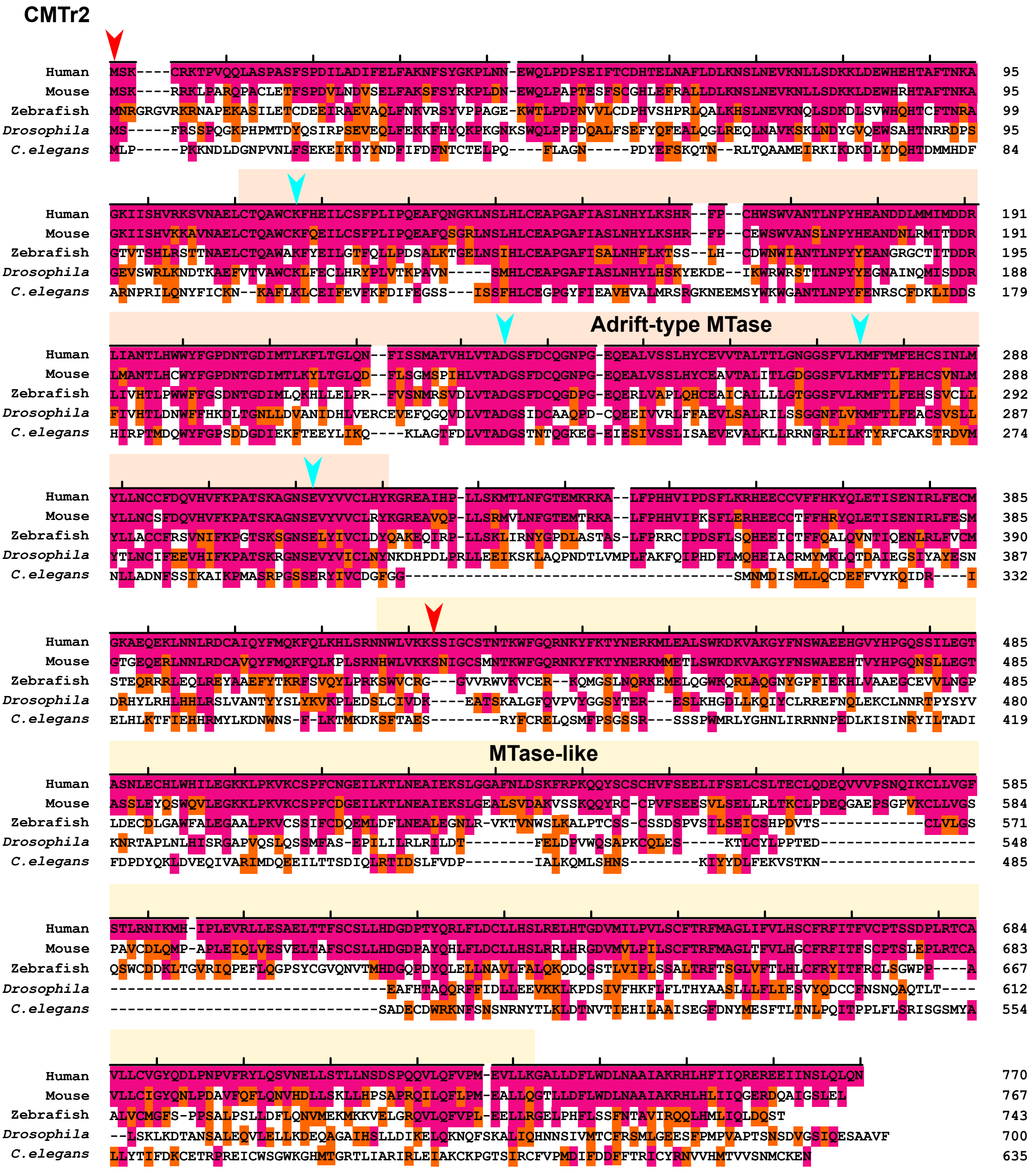

Supplemental Figure S3

PCIF1

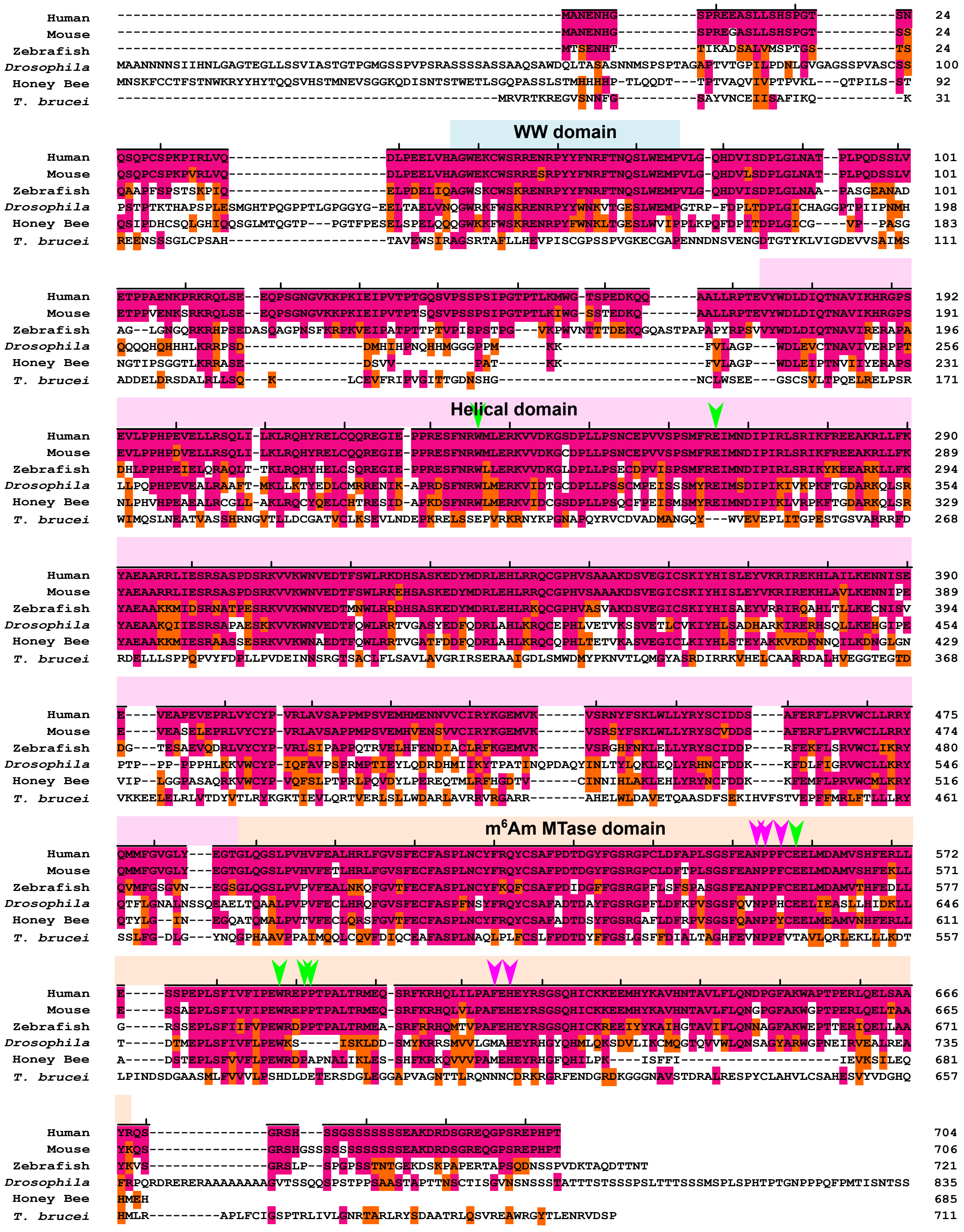

Supplemental Figure S4

A

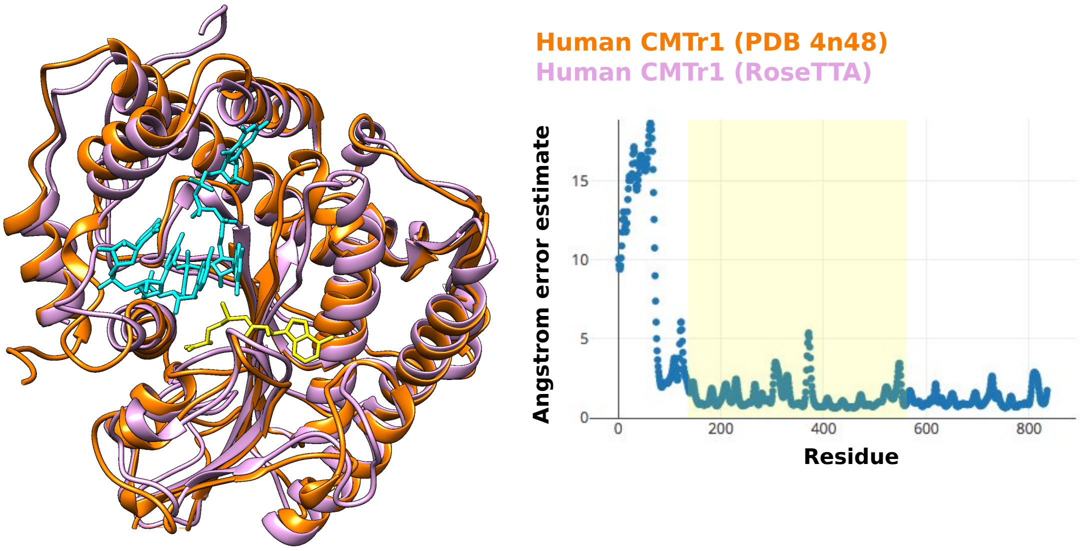

B

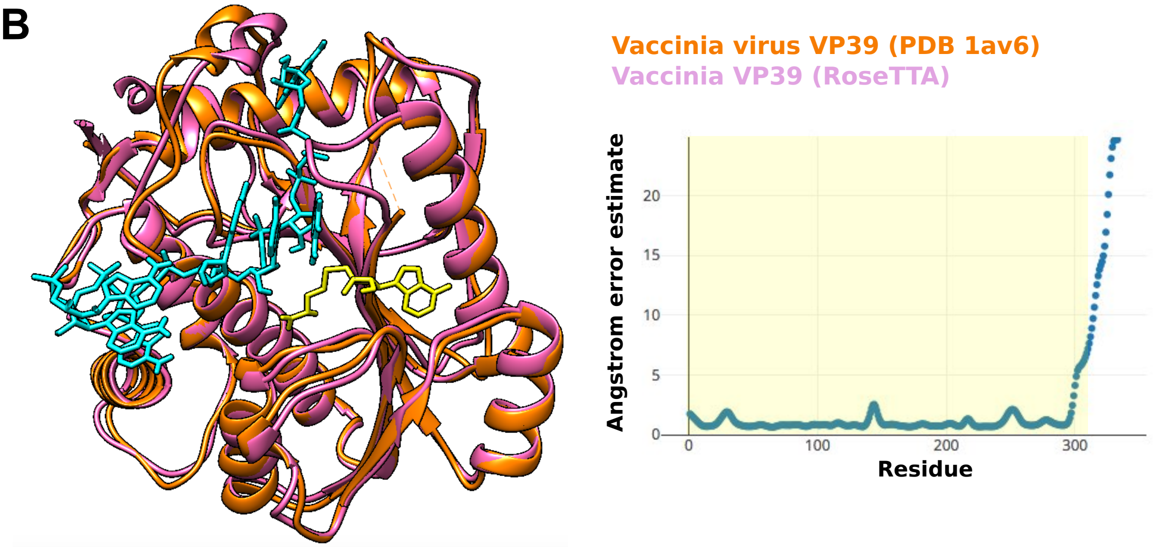

C

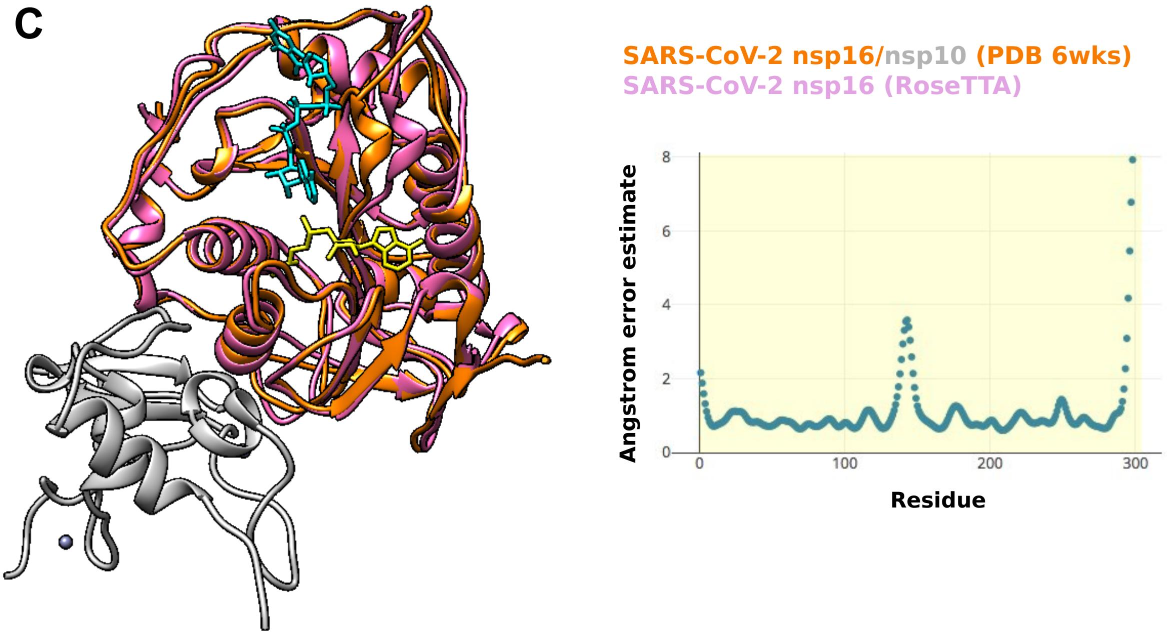

Supplemental Figure S5

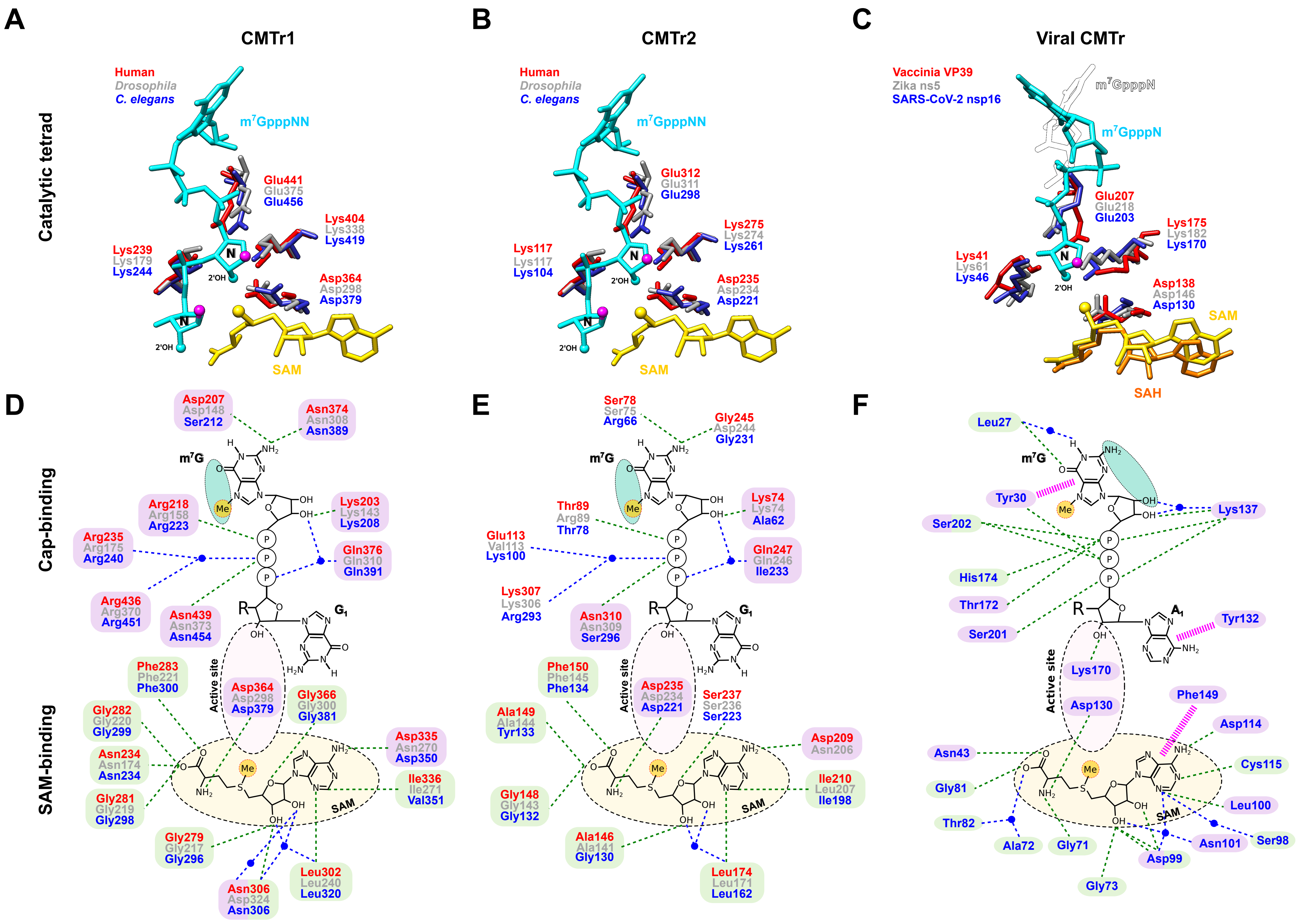

Supplemental Figure S6

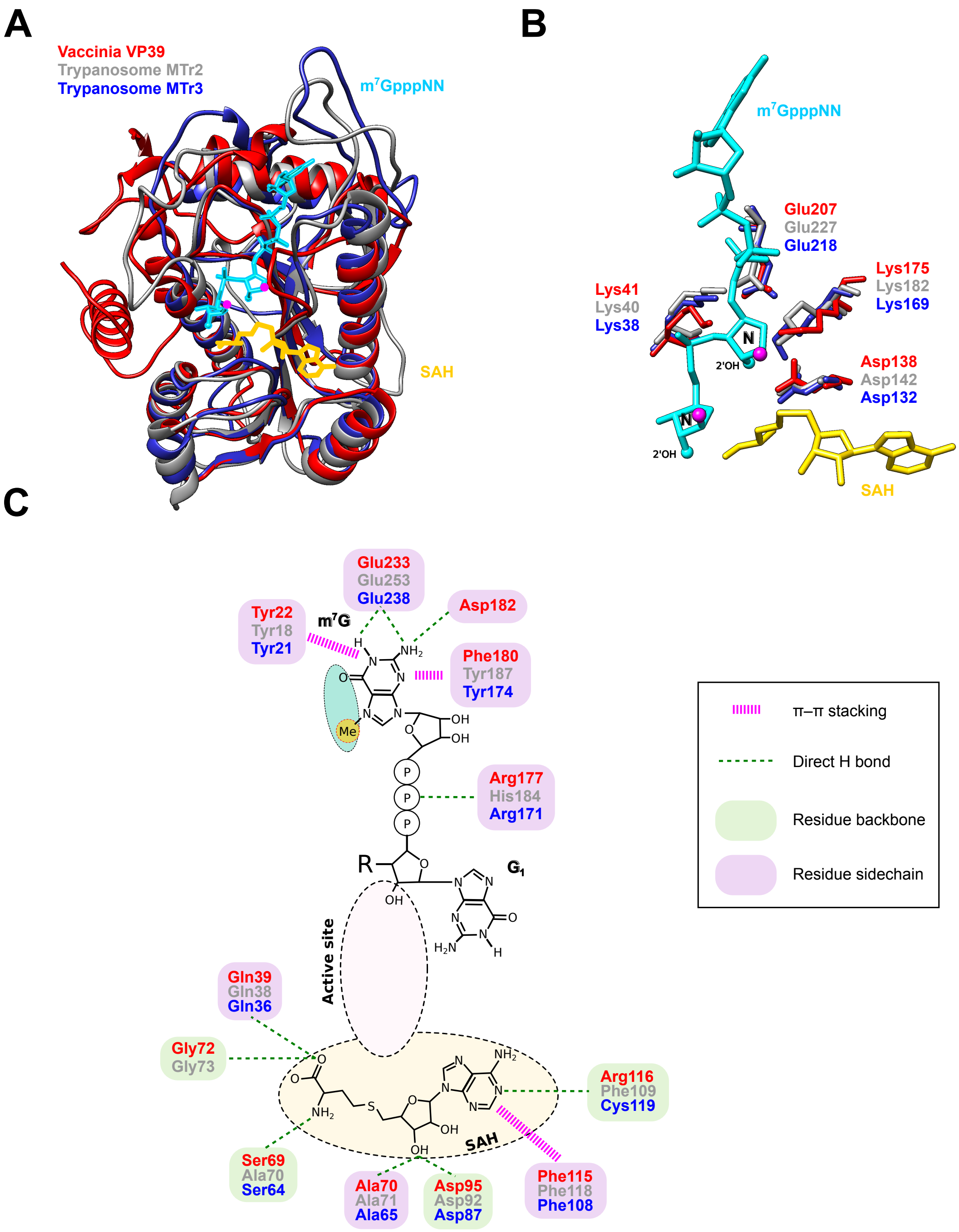

Supplemental Figure S7

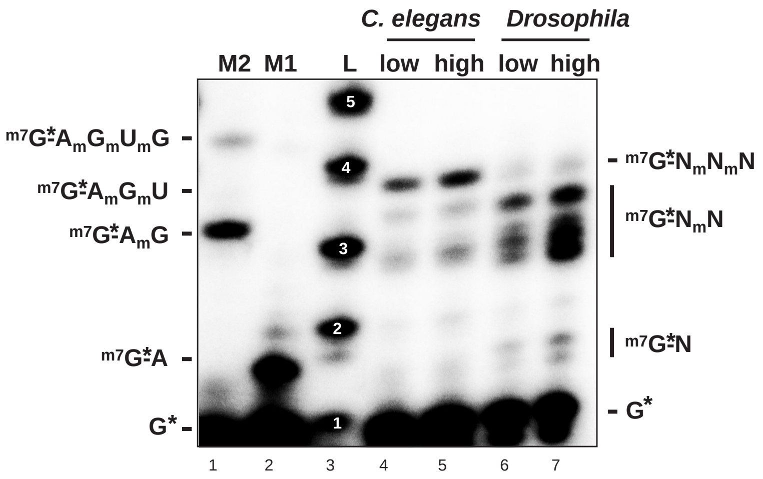

Supplement: Supplemental Material [file supp_079317.122_Supplemental_Material.pdf]
